# Proportion and Associated Factors of Nonreassuring Fetal Heart Rate Patterns in Finote Selam Primary Hospital, North West Ethiopia

**DOI:** 10.1155/2020/6948972

**Published:** 2020-09-19

**Authors:** Eden Asmare Kassahun, Amlaku Mulat Aweke, Almaz Aklilu Getu, Getahun Belay Gela, Simachew Kassa Limenih, Mesafint Ewnetu Mekonnen, Tilksew Ayalew Abtie

**Affiliations:** ^1^Midwifery Department, College of Medicine and Health Sciences, Bahir Dar University, Bahir Dar, Ethiopia; ^2^Nursing Department, College of Medicine and Health Sciences, Bahir Dar University, Bahir Dar, Ethiopia

## Abstract

**Introduction:**

Nonreassuring fetal heart rate patterns (NRFHRP) suggest fetal conciliation or a deteriorating ability to handle the stress of labor. Nearly half of stillbirths occurring worldwide are due to hypoxia which is primarily manifested by NRFHRP. Hence, this study assessed the proportion and associated factors of NRFHRP in the Finote Selam primary hospital, North West Ethiopia.

**Methods:**

An institution-based retrospective cross-sectional study was conducted from March 1 to April 1, 2019, on 364 charts of mothers who gave birth from January 2017 to January 2018 at the Finote Selam primary hospital. A computer-based simple random sampling technique was used to select charts. A secondary data was collected using a structured questionnaire adapted from different literatures. The data was entered and analyzed using Epi Info version 7 and Statistical Package for the Social Sciences (SPSS) version 23.0. Binary logistic regression was executed, and all explanatory variables with *p* value < 0.2 were entered into multivariable logistic regressions. Multivariable logistic regression was used to control the effect of confounding variables and to identify factors affecting NRFHRP. Odds ratios with 95% confidence intervals were computed, and statistical significance was declared if *p* < 0.05.

**Result:**

Out of 364 total deliveries, NRFHRP was detected on 55 (15.1%) fetuses, and the commonest NRFHRP detected was bradycardia 44 (80%). Most NRFHRP (38.18%) occurred on the deceleration phase of labor. There was no identified possible cause for NRFHRP on 34.5% of cases. Referral from nearby health institutions [AOR = 2.832 (95% CI 1.457, 5.503)], primigravida [AOR = 2.722 (95% CI 1.377, 5.381)], augmentation of labor [AOR = 3.664 (95% CI 1.782, 7.534)], and meconium-stained amniotic fluid [AOR = 6.491 (95% CI 3.198, 13.173)] were significantly associated with NRFHRP.

**Conclusion:**

The proportion of NRFHRP is high. Referral from nearby health institutions, primigravida mothers, augmentation of labor, and meconium-stained amniotic fluid were significantly associated with NRFHRP. Implementing a better referral link and close monitoring during follow-up could minimize NHFHRP.

## 1. Introduction

Previously, the abnormal fetal heart rate (FHR) was considered the single most reliable measurement to detect fetal distress; however, this led to frequent improper management and inaccuracies since fetal distress by itself is not well defined, and that is why the concept of the nonreassuring fetal heart rate pattern (NRFHRP) was introduced [1].

According to the three-tier classification, FHR is categorized as Category I: baseline heart rate: 110–160 bpm, variability of 5–25 bpm, and no repetitive decelerations; Category II: indeterminate FHR patterns that are not Category I or III or absence of induced accelerations after fetal stimulation; and Category III: sinusoidal FHR pattern and absent baseline FHR variability followed by recurrent late decelerations, bradycardia, or recurrent variable decelerations [2]. In the context of Ethiopia, NRFHRP during labor and delivery of a term fetus is defined as FHR below 120 beats/minute (bradycardia) or above 160 beats/minute (tachycardia) for 10 minutes or more [3]. In preterm, fetus immaturity of the autonomic nervous system will result in a higher baseline heart rate and reduced variability and less accelerations with less frequent and smaller amplitudes (10 bpm) and for a shorter duration (10 sec) [4].

Intrapartum FHR monitoring is a basic component to assess fetal wellbeing during labor. Even though its false positive rate is approximately 99%, failure to recognize or address a concerning FHR can lead to numerous health risks and devastating injuries such as hypoxia/anoxia, either partial or complete brain damage, cerebral palsy, paralysis, nerve damage, and stillbirth [5]. After all, the target of intrapartum FHR monitoring is to detect fetal hypoxia and metabolic acidosis thereby minimizing operative intervention for simple hypoxia, but to intervene if the condition worsens before it results in tissue damage or fetal death [6].

These days, NRFHRP has become the most common indication for operative deliveries and a signal of morbidities requiring admission to the neonatal intensive care unit which adds extra burden on our society [7, 8]. Studies done across the world revealed that prevalence of NRFHRP ranges between 9.9 and 30.7% [6, 9–12], and antepartum haemorrhage, intrauterine growth restriction, amniotic fluid disorders, maternal medical illnesses, induction/augmentation of labor, use of anaesthesia, and presence of meconium-stained amniotic fluid were some of the risk factors identified to have association with NRFHRP [10, 12–16].

In spite of a development program to improve access and quality of health services, neonatal mortality is still an issue in Ethiopia where 29/1000 neonatal deaths were reported, and the highest loss recorded is in the Amhara region (47/1000) [17]. Majority of the loss was secondary to asphyxia which can be reduced through proper intrapartum follow-up. The presence of NRFHRP therefore alarms health professionals about the fetal condition to give proper intervention and prevent morbidities and mortalities of the newborn. Hence, this study intended to assess the proportion and associated factors of NRFHRP in the Finote Selam primary hospital, North West Ethiopia.

## 2. Method

### 2.1. Study Area and Period

The study was conducted from March 1 to April 1, 2019, at Finote Selam town which is one of the five town administrations in the West Gojjam zone. Finote Selam is located 378 km away from Addis Ababa and 176 km away from Bahir Dar. It has 17 health-related institutions, i.e., one governmental district hospital, one health center, two health posts, five private clinics, two pharmacies, and six drug stores. The hospital has four laboring beds, four delivery couches, two functional cardiotocography (CTG) machines, and one integrated neonatal intensive care unit. Around 3,000 births were attended by the year 2018 including caesarean section and instrumental delivery. There were 22 midwives (7 diploma and 15 degree holders), 3 emergency integrated surgical officers, and one senior gynaecologist during the study period.

### 2.2. Study Design and Population

An institution-based retrospective cross-sectional study was conducted. All fully documented and randomly selected 364 charts of women who gave birth at the Finote Selam primary hospital from January 2017 to January 2018 were the study subjects. All randomly selected charts of mothers that had important fetal and maternal parameters were included, and the charts of mothers who had intrauterine fetal death at time of admission, mothers who gave birth at other institutions, and charts that were not fully documented with important parameters were excluded.

### 2.3. Sample Size Calculation and Sampling Procedure

The sample size was determined using a single population proportion formula with the assumptions of 95% confidence interval, marginal error (*d*) of 4%, and prevalence of nonreassuring fetal heart rate patterns (NRFHRP) of 18.6% [6], making the final sample size 364. All 2,978 delivery charts of mothers who gave birth from January 2017 to January 2018 were collected. Charts that were fully documented with the important fetal and maternal parameters were identified. 2,002 charts that had important fetal and maternal parameters were selected and given codes based on their order of registration number. A computer-based simple random sampling technique was used to select 364 charts.

### 2.4. Study Variables

The dependent variable of the study was NRFHRP (yes/no), and the independent variables were sociodemographic characteristics (maternal residence, age, and marital status) antepartum findings (suspected or confirmed fetal growth restriction, amniotic fluid disorders, being primigravida, antepartum haemorrhage, and preeclampsia), and intrapartum findings (referral from outset, oxytocin induction/augmentation, abnormal vaginal bleeding in labor, and epidural block).

### 2.5. Data Collection Tool and Procedure

A structured and pretested questionnaire adapted from different literatures related to the topic was used. The questionnaire was segmented into three parts. The first part tried to assess sociodemographic characteristics of mothers (residence, age, and marital status). The second part assessed the antepartum profile of mothers (ANC follow-up, number of pregnancies, type of pregnancy, ultrasound finding, maternal medical illness, bad obstetric history, haematocrit, and blood group of mothers), and the third part assessed the intrapartum profile of mothers and fetuses (way of admission, gestational age, presentation of fetus, onset of labor, duration of labor, FHR on admission, stage of labor on admission, stage of labor at detection of NRFHRP, and intrapartum complication). Data was collected by two diploma holder nurses and supervised by one BSc nurse. Training about the data collection procedure and supervision was given for a day before data collection. A pretest was performed on 10% of the study participants at Dangala district hospital two weeks prior to the actual data collection. Data quality was ensured during collection, coding, entry, and analysis. Supervision including observation of how the data was collected was done by the supervisor. The filled questionnaires were checked for completeness by data collectors, the supervisor, and the principal investigator on a daily basis. Consequently, any problem encountered was discussed among the team and solved immediately.

### 2.6. Data Processing and Analysis

The data were coded and entered to EpiData version 3.1 and analyzed by Statistical Package for the Social Sciences (SPSS) version 23.0. Bivariate analysis was performed to assess the potential associations between categorical variables and the outcome. Variables with *p* value of less than 0.2 in the bivariate analysis were entered into the final multivariable logistic regression model to identify the predictors of the outcome. Odds ratio with 95% confidence intervals were computed, and statistical significance was declared if *p* < 0.05.

### 2.7. Operational Definitions

- *Normal FHR pattern*: baseline FHR of 120-160 beats/min for a term fetus [3]
- *Abnormal FHR pattern*: baseline FHR < 120 or >160
- *Bradycardia*: baseline FHR < 120
- *Tachycardia*: baseline FHR > 160
- *NRFHRP*: abnormal FHR recorded more than twice during intrapartum follow-up
- *Primigravida*: an individual pregnant for the first time

## 3. Results

### 3.1. Sociodemographic Characteristics

Most mothers (175 (48.2%)) were found in the age category of 15-25, and the range was from 17 to 42 years of age. The mean age was 26.75 ± 5.605. Around 186 (51.2%) mothers reside in the urban area. More than half of the mothers (243(66.7%)) were married (Table 1).

### 3.2. Antepartum Profile of Mothers

Among mothers who were admitted, 37 (10.2%) had at least one antenatal care (ANC) visit. Mothers who had ultrasound scan during the ANC follow-up or after admission were 121 (33.3%). There were 154 (42.4%) primigravida mothers. Most women (323 (88.7%)) had a normal haematocrit level. Around 45 (12.4%) mothers had medical illness, where the most frequently encountered illness was urinary tract infection (4.4%). Mothers who had bad obstetric history were 49 (13.5%) and half of them (25 (51.02%)) had no specified documentation on the type. The blood group of most mothers was 126 (34.6%) O+ (Table 2).

### 3.3. Intrapartum Profile of Mothers

Out of total admissions, 134 (37.2%) mothers were referred from other health facilities. On admission, about 316 (87.05%) mothers had spontaneous onset of labor. The mean gestational age was 37.9 ± 1.6 weeks. Among mothers who had spontaneous labor, 66 (18.13%) had augmentation. Majority of the babies, that is, 356 (97.8%), were singleton, and 333 (91.5%) of them were in cephalic presentation. Around 89 (24.5%) mothers received medication where the most frequently administered medication was antibiotic. Nearly half of the mothers (171 (46.97%)) were admitted in the latent first stage of labor, and most of the nonreassuring fetal heart rate patterns (NRFHRP) occurred in the deceleration phase (38.18%). Duration of labor lasted from 0 to 30 hrs, making its mean 7.536 ± 4.2 hrs. Mothers admitted with a detected NRFHRP were 24 which made the prevalence on admission 6.6%. Meconium-stained amniotic fluid was seen on 131 (35.8%) of the delivered babies. There was no identified possible complication to cause NRFHR in 19/55 (34.5%) deliveries (Table 3).

### 3.4. Proportion of NRFHRP

The overall proportion of NRFHRP was 55 (15.1%). The most common FHR abnormality detected was bradycardia (44 (80%)), and tachycardia was recorded in the remaining 11/55 (20%).

### 3.5. Associated Factors of NRFHRP

Six variables (residence, maternal medical illness, being primigravida, meconium-stained amniotic fluid, augmentation of labor, and referral from other health facilities) had shown significant association during bivariate analysis. However, during multiple variable analysis, referral from other health facilities [AOR = 2.832 (95% CI 1.457, 5.503)], being primigravida [AOR = 2.722 (95% CI 1.377, 5.381)], augmentation of labor [AOR = 3.664 (95% CI 1.782, 7.534)], and meconium-stained amniotic fluid [AOR = 6.491 (95% CI 3.198, 13.173)] showed significant association (Table 4).

## 4. Discussion

The proportion of nonreassuring fetal heart rate pattern (NRFHRP) in this study was found to be 15.1% (95% CI 11.6%-19.2%) which is in line with the cross-sectional study conducted in Addis Ababa (18.6%) [6]. The proportion is higher than findings from the randomized controlled trial of Tanzania (9.9%) [11] and case control study done in Zimbabwe Harare (11.2%) [15]. This might be due to the difference in methodology. In the Tanzanian study, gestational age > 37 weeks, cephalic presentation, and cervical dilation < 7 cm were considered as inclusion criteria, and mothers with placental abruption, uterine rupture, and multiple pregnancy were excluded. In the study from Zimbabwe Harare, only term babies in cephalic presentation and a normal fetal heart beat on admission were assessed. On the other hand, it was lower than findings from Thailand (30.7%) [12] and Israel (21.1%) [14]. This might be due to the wide use of CTG monitoring in the developed world. It may also be due to the wider classification of NRFHRP in the other studies which is limited to bradycardia and tachycardia in this study.

According to the analysis, certain antepartum and intrapartum factors were found to be associated with NRFHRP. Mothers referred from other health facilities were nearly 3 times more likely to develop NRFHRP than mothers who were directly admitted to the hospital. This finding is also mentioned in another study [18]. The cause might be a delay in transportation. Missing conservative measures as intrauterine resuscitation before or during referral of patients with risk factors may also be the reason. It might also be due to poor health-seeking behaviour of mothers getting service at other facilities especially those mothers referred from rural health centers.

Primigravida women were nearly 3 times more likely to have NRFHRP than multigravida mothers. This association is also supported by studies conducted in India [16] and Thailand [12]. Being primigravida is a high-risk pregnancy with several associated obstetric complications such as preeclampsia, prolonged duration of labor, and other conditions which deprive both maternal and fetal optimal conditions leading to disturbed fetal heart rate patterns [19]. A primigravida mother is new to the whole course of labor and pregnancy leading her to anxiety and stress which causes physiologic disruption of blood flow to the fetus resulting uterine hypoxia [20].

On the other hand, this finding contrasts with the case control study conducted in Israel [21] where being primigravida decreased the risk of developing NRFHRP. This might be due to proper ANC counselling as many of the participants in the Israel study had ANC follow-up. They may have awareness on danger signs of pregnancy or labor that could compromise fetal conditions.

Mothers who had augmentation of labor were 3 times more likely to develop NRFHRP than mothers who had no augmentation. In a similar manner, a study conducted in Israel showed that oxytocin augmentation significantly increased the risk for NRFHRP [21]. The administration of oxytocin to correct uncoordinated labor patterns confers a risk for increase in uterine contraction frequency and decrement of uterine blood flow which causes hypoxemia and disturbs the FHR pattern [22]. Regardless of the presence of clear indication, lost intrapartum follow-up of a mother during augmentation could trigger NRFHRP. It may also be due to a psychological disturbance of the mother due to the mere concept of being medicated. A probable suboptimal condition of both the mother and the fetus due to prolonged labor may also be a contributing factor.

Fetuses which had meconium-stained amniotic fluid were 6 times more likely to develop NRFHRP than fetuses that had clear amniotic fluid, which is similar with other studies [14, 16]. FHR abnormalities are first indicators of fetal hypoxic stress which then stimulate colonic activity, by enhancing intestinal peristalsis and relaxing the anal sphincter resulting in passage of meconium [20]. However, this is contrary to the report from the case control study conducted in India [9] which found no significant association between meconium-stained liquor and NRFHRP. This may be due to the difference in methodology where cases selected in the India study were only term fetuses in cephalic presentation, but in this study, fetuses that were in breach presentation and postterm were also included.

The study had limitations such as missing vital information and clinician differences on interpretation of NRFHRP; however, proper training was given for data collectors to minimize these biases. Hence, this study could be insightful towards the proportion and associated factors of NRFHRP.

## 5. Conclusion and Recommendation

The proportion of NRFHRP was high compared to studies conducted in other African countries. Bradycardia was more frequently seen among fetuses having nonreassuring fetal heart rate patterns. Most of the NRFHRP occur during the deceleration phase, and the majority had no clear identifiable cause. Referral from other health facilities, being primigravida, augmentation of labor, and meconium-stained amniotic fluid were factors that showed significant association with NRFHRP. It would be better if nearby health institutions give more attention on the ANC follow-up of mothers, way of referring, and documentation of information as most of the mothers who were referred had either no ANC follow-up or no documentation of their vital findings. As meconium-stained amniotic fluid showed strong association, giving due attention to its presence and anticipating NRFHRP may prevent further complications. During augmentation of labor, it is better if health care providers consider its possible complications and monitor closely. Health care providers should consciously follow primigravida mothers and give them adequate information about the nature of labor, psychological support, and possible intrapartum complications they may face.

## Figures and Tables

**Table 1:** Sociodemographic characteristics of mothers in Finote Selam primary hospital, North West Ethiopia, 2019 (*n* = 364).

Variables	Frequency	Percentage
Age		
15-25	175	48.2
26-35	164	44.9
36-45	25	6.9
Residence		
Urban	186	51.2
Rural	178	48.8
Marital status		
Married	243	66.7
Single	17	4.7
Widowed	2	0.5
Not documented	102	28.1

**Table 2:** Antepartum profile of mothers who gave birth at the Finote Selam primary hospital, North West Ethiopia, 2019.

Variables	Frequency	Percentage
ANC follow-up		
0	8	2.2
1	37	10.2
2	63	17.3
3	110	33
4	76	21.9
No documentation	57	15.7
Gravidity		
Multigravida	210	57.6
Primigravida	154	42.4
Type of pregnancy		
Single	356	97.8
Multiple	8	2.2
Ultrasound index		
Abnormal pregnancy	34	9.3
Normal pregnancy	108	29.5
No documentation	222	61.2
Maternal medical illness (*n* = 45)		
Hypertensive disorders of pregnancy	6	1.65
Diabetes mellitus	4	1.1
Malaria	9	2.5
Urinary tract infection	18	4.9
Asthma	3	0.82
Others^∗^	5	1.4
Haematocrit level		
In the normal range	323	88.7
Anaemic	41	11.3
Type of bad obstetric history (*n* = 49)		
Early neonatal loss	10	20.4
Stillbirth	12	24.4
Ectopic pregnancy	2	4.08
Not specified	25	51.02
Maternal blood group		
O-	30	8.3
O+	126	34.6
A-	15	4.1
A+	98	27
B-	14	3.9
B+	54	14.9
AB-	3	0.82
AB+	24	6.6

^∗^Hepatitis, tuberculosis, and HIV.

**Table 3:** Intrapartum profile of mothers who gave birth at Finote Selam primary hospital North West Ethiopia, 2019.

Variables	Frequency	Percentage
Gestational age		
<37 weeks	23	6.3
37-42 weeks	335	92
>42 weeks	6	1.65
Type of pregnancy		
Single	356	97.8
Multiple	8	2.2
Onset of labor		
Spontaneous	317	87.05
Induced	41	11.3
Elective C/S	6	1.65
Presentation of the baby		
Cephalic	333	91.5
Breech	24	6.6
Transverse	3	0.82
Compound	4	1.1
Stage of labor on admission		
Closed	23	6.3
1-3 cm	171	46.8
4-9 cm	145	39
10 cm	25	6.9
FHR on admission		
Normal	340	93.4
Abnormal	24	6.6
Stage of labor at detection of NRFHRP (*n* = 55)		
Closed	2	3.6
1-3 cm	14	25.5
4-9 cm	18	32.7
10 cm	21	38.18
Degree of meconium staining		
Clear	233	64
Grade I	88	24.24
Grade II	32	8.8
Grade III	11	3.03
Duration of labor		
Prolonged	72	19.7
Not prolonged	292	80.3
Possible cause for NRFHRP (59)		
Unknown cause	19	34.2
Poor progress of labor	12	21.8
Fetopelvic disproportion	11	20
Nuchal cord	5	9.1
Chorioamnionitis	5	9.1
Antepartum haemorrhage (placental abruption)	3	5.45
Preeclampsia	3	5.45
Previous C/S scar	2	0.55
Cord prolapse	1	1.81

**Table 4:** Bivariate and multivariate analyses of factors associated with nonreassuring fetal heart rate patterns at Finote Selam primary hospital, North West Ethiopia, 2019.

Variable	NRFHRP				
	Yes (%)	No (%)	COR (95% CI)	AOR (95% CI)	*p* value
Residence					
Urban	32 (17.2)	154 (82.8)	1		
Rural	23 (13.1)	155 (86.9)	1.40 (0.339, 2.102)	1.57 (0.767, 3.001)	0.44
Gravidity					
Primigravida	38 (24.7)	116 (75.3)	3.72 (1.997, 6.850)	2.72 (1.377, 5.381)	0.04^∗^
Multigravida	17 (8.1)	193 (91.9)	1		
Referral					
Yes	31 (23.1)	103 (76.9)	2.58 (1.680, 5.460)	2.83 (1.457, 5.503)	0.02^∗^
No	24 (10.4)	206 (89.6)	1		
Augmentation of labor					
Yes	23 (34.8)	43 (65.2)	4.45 (2.469, 8.776)	3.66 (1.782, 7.534)	0.001^∗^
No	32 (10.7)	266 (89.3)	1		
Meconium staining					
Yes	42 (32.1)	89 (67.9)	7.98 (4.072, 15.52)	6.49 (3.198, 13.173)	0.001^∗^
No	13 (5.6)	220 (94.4)	1		
Maternal medical illness					
Yes	6 (13.3)	39 (86.7)	0.85 (0.339, 2.102)	0.77 (0.389, 1.509)	0.45
No	49 (15.4)	270 (84.6)	1		

^∗^ *p* < 0.05, significant.

## Data Availability

The data that support the findings of this study are available, which were used under permission for the current study, but not publicly available. The data are however available from the authors upon reasonable request.

## Abbreviations

ANC: Antenatal care
CS: Caesarean section
CTG: Cardiotocography
EC: Ethiopian calendar
FHR: Fetal heart rate
NRFHRP: Nonreassuring fetal heart rate patterns
SPSS: Statistical Package for the Social Sciences.

## References

[1] *Fetal distress: diagnosis, conditions & treatment*.

[2] Santo S., Da grac a1 for the fm-compare collaboration (2016). *Agreement and accuracy using the FIGO, ACOG and NICE cardiotocography interpretation guidelines, Cristina Costa-Santos, William Schnettler, Austin Ugwumadu & Lu is diotocography interpretation guidelines*.

[3] Ethiopia F. (2010). *Labor and delivery care*.

[4] (2018). *Intrapartum fetal monitoring guideline*.

[5] Fuller A. (2017). *Intrauterine fetal assessment of non-reassuring status*.

[6] Kassa E. M. (2018). *Clinical profile and out come of pregnancies with NRFHR in labor at three teaching hospitals*.

[7] Hubena Z., Workneh A., Siraneh Y. (2018). Prevalence and outcome of operative vaginal delivery among mothers who gave birth at Jimma University Medical Center, Southwest Ethiopia. *Journal of Pregnancy*.

[8] Abebe F. E., Gebeyehu A. W., Kidane A. N., Eyassu G. A. (2018). Factors leading to cesarean section delivery at Felegehiwot referral hospital, Northwest Ethiopia: A retrospective record review. *Reproductive Health*.

[9] Desai D., Chauhan K., Chaudhary S. (2013). A study of meconium stained amniotic fluid, its significance and early maternal and neonatal outcome. *International Journal of Reproduction, Contraception, Obstetrics and Gynecology*.

[10] Ganer Herman H., Tamayev L., Houli R., Miremberg H., Bar J., Kovo M. (2018). Risk factors for nonreassuring fetal heart rate tracings after artificial rupture of membranes in spontaneous labor. *Birth*.

[11] Mdoe P. F., Ersdal H. L., Mduma E. R. (2018). Intermittent fetal heart rate monitoring using a fetoscope or hand held Doppler in rural Tanzania: a randomized controlled trial. *BMC Pregnancy and Childbirth*.

[12] Boonchuan K., Wattananirun K., Boriboonhirunsarn D. (2018). Incidence of Intrapartum abnormal fetal heart rate pattern in Siriraj hospital. *Thai Journal of Obstetrics and Gynaecology*.

[13] Xu H., Mas-Calvet M., Wei S.-Q., Luo Z.-C., Fraser W. D. (2009). Abnormal fetal heart rate tracing patterns in patients with thick meconium staining of the amniotic fluid: association with perinatal outcomes. *American Journal of Obstetrics and Gynecology*.

[14] Hadar A., Sheiner E., Hallak M., Katz M., Mazor M., Shoham-Vardi I. (2001). Abnormal fetal heart rate tracing patterns during the first stage of labor: effect on perinatal outcome. *American Journal of Obstetrics and Gynecology*.

[15] Desai D., Maitra N., Patel P. (2017). Fetal heart rate patterns in patients with thick meconium staining of amniotic fluid and its association with perinatal outcome. *International Journal of Reproduction, Contraception, Obstetrics and Gynecology*.

[16] Bhatia N., Krishna K. M. Intraoperative findings in primary caesarean section for non-reassuring fetal status and its correlation with cardiotocography. *International Journal of Reproduction, Contraception, Obstetrics and Gynecology*.

[17] (2016). *Ethiopia Demographic and Health Survey*.

[18] Ajah L. O., Ibekwe P. C., Onu F. A., Onwe O. E., Ezeonu T. C., Omeje I. (2016). Evaluation of clinical diagnosis of fetal distress and perinatal outcome in a low resource Nigerian setting. *Journal of Clinical and Diagnostic Research: JCDR*.

[19] Okunade K. S., Okunola H., Oyeneyin L., Habeeb-Adeyemi F. N. (2016). Cross-sectional study on the obstetric performance of primigravidae in a teaching hospital in Lagos, Nigeria. *Nigerian Medical Journal: journal of the Nigeria Medical Association*.

[20] Hashim N., Naqvi S., Khanam M., Jafry H. F. (2012). Primiparity as an intrapartum obstetric risk factor. *JPMA-Journal of the Pakistan Medical Association*.

[21] Ganer Herman H., Tamayev L., Houli R., Miremberg H., Bar J., Kovo M. (2018). *Risk factors for non-reassuring fetal heart rate tracings after artificial rupture of membranes in spontaneous labor*.

[22] Sletten J., Kiserud T., Kessler J. (2016). Effect of uterine contractions on fetal heart rate in pregnancy: a prospective observational study. *Acta obstetricia et Gynecologica Scandinavica*.

